# Development of Functional Microfold (M) Cells from Intestinal Stem Cells in Primary Human Enteroids

**DOI:** 10.1371/journal.pone.0148216

**Published:** 2016-01-28

**Authors:** Joshua D. Rouch, Andrew Scott, Nan Ye Lei, R. Sergio Solorzano-Vargas, Jiafang Wang, Elaine M. Hanson, Masae Kobayashi, Michael Lewis, Matthias G. Stelzner, James C. Y. Dunn, Lars Eckmann, Martín G. Martín

**Affiliations:** 1 Department of Surgery, Division of Pediatric Surgery, David Geffen School of Medicine at UCLA, University of California Los Angeles, Los Angeles, California, United States of America; 2 Department of Bioengineering, Henry Samueli School of Engineering, University of California Los Angeles, Los Angeles, California, United States of America; 3 Department of Pediatrics, Division of Gastroenterology and Nutrition, Mattel Children’s Hospital and the David Geffen School of Medicine at UCLA, University of California Los Angeles, Los Angeles, California, United States of America; 4 Department of Medicine, University of California San Diego, La Jolla, California, United States of America; 5 Department of Pathology, Veterans Affairs Greater Los Angeles Healthcare System, Los Angeles, California, United States of America; 6 Department of Surgery, Veterans Affairs Greater Los Angeles Healthcare System, Los Angeles, California, United States of America; 7 Eli and Edythe Broad Center of Regenerative Medicine & Stem Cell Research, University of California Los Angeles, Los Angeles, California, United States of America; University of Kentucky, UNITED STATES

## Abstract

**Background & Aims:**

Intestinal microfold (M) cells are specialized epithelial cells that act as gatekeepers of luminal antigens in the intestinal tract. They play a critical role in the intestinal mucosal immune response through transport of viruses, bacteria and other particles and antigens across the epithelium to immune cells within Peyer’s patch regions and other mucosal sites. Recent studies in mice have demonstrated that M cells are generated from Lgr5+ intestinal stem cells (ISCs), and that infection with *Salmonella enterica* serovar Typhimurium increases M cell formation. However, it is not known whether and how these findings apply to primary human small intestinal epithelium propagated in an *in vitro* setting.

**Methods:**

Human intestinal crypts were grown as monolayers with growth factors and treated with recombinant RANKL, and assessed for mRNA transcripts, immunofluorescence and uptake of microparticles and *S*. Typhimurium.

**Results:**

Functional M cells were generated by short-term culture of freshly isolated human intestinal crypts in a dose- and time-dependent fashion. RANKL stimulation of the monolayer cultures caused dramatic induction of the M cell-specific markers, *SPIB*, and Glycoprotein-2 (*GP2*) in a process primed by canonical WNT signaling. Confocal microscopy demonstrated a pseudopod phenotype of GP2-positive M cells that preferentially take up microparticles. Furthermore, infection of the M cell-enriched cultures with the M cell-tropic enteric pathogen, *S*. Typhimurium, led to preferential association of the bacteria with M cells, particularly at lower inoculum sizes. Larger inocula caused rapid induction of M cells.

**Conclusions:**

Human intestinal crypts containing ISCs can be cultured and differentiate into an epithelial layer with functional M cells with characteristic morphological and functional properties. This study is the first to demonstrate that M cells can be induced to form from primary human intestinal epithelium, and that *S*. Typhimurium preferentially infect these cells in an *in vitro* setting. We anticipate that this model can be used to generate large numbers of M cells for further functional studies of these key cells of intestinal immune induction and their impact on controlling enteric pathogens and the intestinal microbiome.

## Introduction

The single layer of epithelial cells that lines the entire intestinal tract is the primary physical barrier separating the intestinal lumen and its content from the intestinal lamina propria and the body’s interior. Various mechanisms have been proposed to explain enteral uptake of viruses and microbes, including disruptions of the epithelial barrier, transcytosis across enterocytes, infection of juxtaposed dendritic cells and/or lymphocytes, and through microfold (M) cells located within the follicle-associated epithelium (FAE) that overlies Peyer’s patches [1], or are scattered along the villus-independent of Peyer’s patches [2]. These cells represent a major site for the sampling of gut luminal antigens, and are important for enteral uptake of various commensal microorganisms, and viral and bacterial pathogens including *Salmonella enterica* serovar Typhimurium, *Vibrio cholerae*, HIV, Reovirus, Poliovirus and others [1–4].

M cells lack the typical tightly packed long microvilli characteristic of enterocytes, and instead have disorganized stubby microvilli along their apical border [2]. In addition, M cells have deep basolateral invaginations that are often found to harbor immune cells. This morphology allows for efficient antigen and microbial sampling along with rapid transcytosis. Thus far, the assessment of how various pathogens are transmitted across the human intestinal epithelial layer has been limited to immortalized colon cancer cell lines [5–7].

All epithelial cells of the gut are derived from dividing crypt-based intestinal stem cells (ISCs) that undergo long-term self-renewal to maintain the stem cell population and are uniquely identified by expression of *Lgr5* [8]. The differentiated epithelial lineages of the small intestine include Paneth cells, goblet cells, tuft cells, enterocytes, enteroendocrine cells, and M cells, all of which have specialized roles required for proper alimentation within the context of a complex symbiotic luminal microbiota. By nature, the ISCs are particularly sensitive to injury under stressful conditions including infection; however, a quiescent cell population of secretory progenitor cells (+4 cells) can de-differentiate into *bona fide* LGR5+ rapidly dividing ISCs [9]. Under both homeostatic and stressed conditions, the function of ISCs and the quiescent +4 cell are believed to be influenced by various cells within the niche–including subepithelial myofibroblasts, lymphocytes, macrophages, and dendritic cells.

Epithelial lineage differentiation in the small intestine is controlled a complex cascade of lineage-specific transcription factors that are activated by the Notch signaling pathway in ISCs and early progenitor cells which dictates differentiation into absorptive or secretory cell lineages [10–12]. Furthermore, lineage differentiation is influenced by the cellular microenvironment. For example, development of M cells located within the FAE is controlled in mice by a subepithelial network of reticular cells and B cells that secrete the cytokine, receptor activator of NF-κB ligand (RANKL)—a type II member of the tumor necrosis factor superfamily [13]. The binding of RANKL to its receptor, RANK (TNFRSF11a), activates the non-canonical (RelB) NF-κB signaling pathway, and induces the expression of *SpiB*, an ETS-transcription factor that drives M cell fate determination and maturation [14–16].

Recent studies have demonstrated that M cells can be generated using a murine *in vitro* enteroid model in a RANKL-dependent manner [17]. Lineage-tracing studies demonstrated that M cells are derived from LGR5+ ISCs through RANKL induction of *SpiB*. RANKL stimulation of LGR5+ crypts from *SpiB* null mice failed to generate M cells, confirming that *SpiB* expression is required for M cell development [17]. In addition, acute *S*. Typhimurium infection in mice has been shown to induce rapid transdifferentiation of enterocytes into functional M cells through WNT/**β**-catenin and NF-κB dependent epithelial mesenchymal transition (EMT) [16]. This follows similar patterns of EMT observed in other cell types through the same WNT/**β**-catenin and NF-κB signaling pathways [18–20].

Intestinal enteroids are an *in vitro* model that supports the proliferation and differentiation of ISCs into the full array of epithelial lineages that comprise the lining of the gut [8]. Several groups, including our own, have developed methods to grow human enteroids including in a 2D modular configuration using Transwells that separates luminal and subepithelial compartments [21,22]. Here we set out to adapt the enteroid model for the formation of M cells from proximal human small intestinal crypts, and characterize the cells in regard to their ability to endocytose microparticles and permit uptake of *S*. Typhimurium, both of which are characteristics of M cells *in vivo*.

## Results

### RANKL-Induces M Cell Differentiation in a Dose- and Time-Dependent Fashion

**Fig 1:**
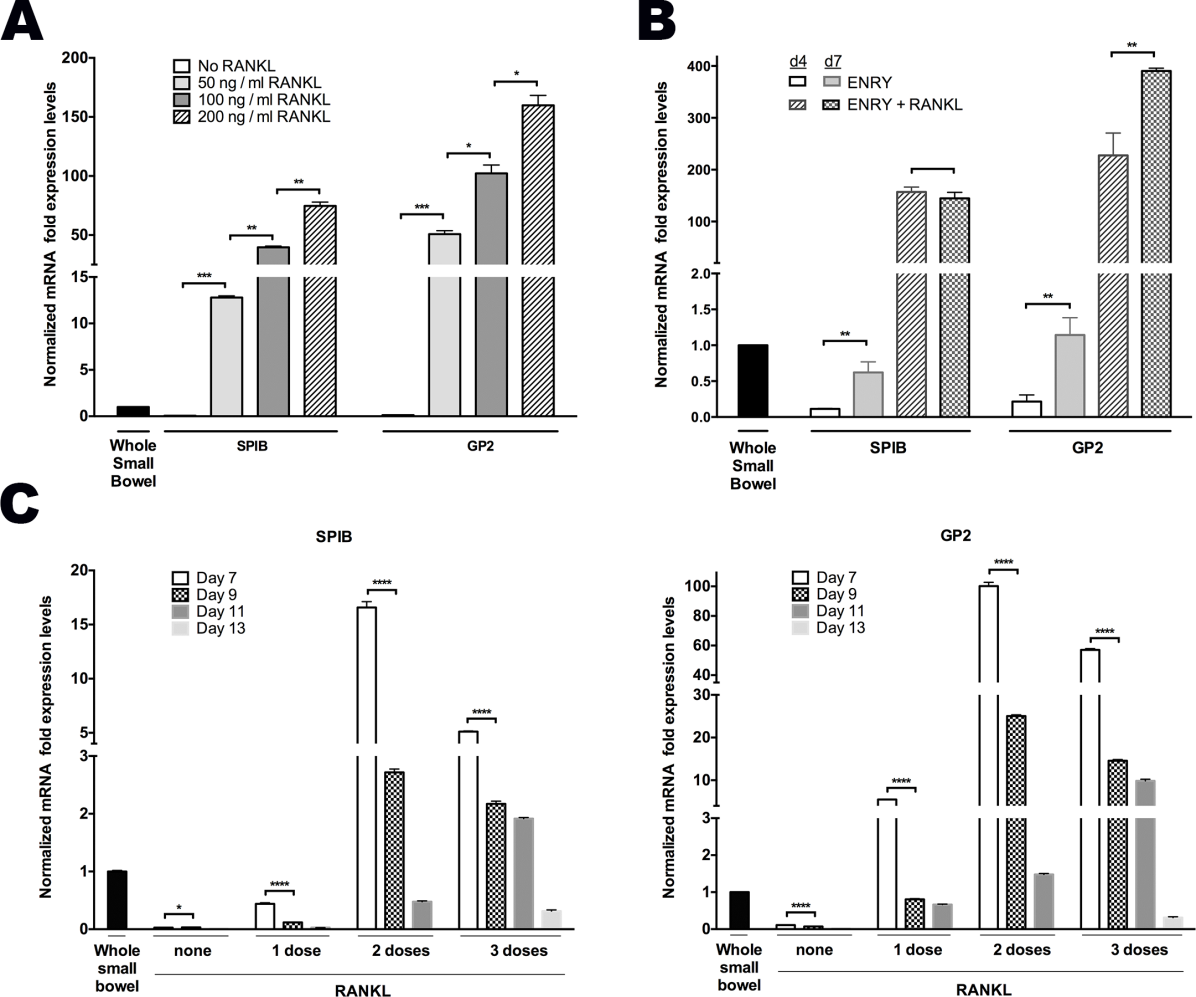
RANKL induces M cell differentiation in a dose and time dependent manner. **(A)** Dose-dependent increases of *SPIB* and *GP2* mRNA expression in 50, 100 and 200 ng/mL RANKL and ENRY treated monolayers assessed at day 5. **(B)** Time course of *SPIB* and *GP2* peaked expression levels in monolayers treated with and without 200ng/mL RANKL. **(C)** Time decay of *SPIB* and *GP2* expression among monolayers receiving 0, 1, 2, or 3 doses of RANKL every other day from day 0, and assessed on days 7, 9, 11 and 13. All results normalized to *GAPDH* expression in human whole proximal small bowel or crypts, as indicated. *p<0.*05*, **p<0.*01*, ***p<0.*001*, ****p<0.*0001*.

Primary human intestinal crypts isolated from discarded surgical samples of the proximal small intestine were grown on both 2D collagen-coated plates, and as 3D Matrigel plugs in ENRY medium that promotes their growth [22,23] with and without addition of recombinant RANKL every other day. RANKL stimulated a marked increase in *SPIB* expression at each concentration tested in 2D-grown monolayers when compared to proximal whole small bowel controls [Fig 1A]. Similarly, RANKL promoted expression of *GP2*, a cell surface marker specifically expressed on M cells and absent on other ISC-derived cells [24] in a concentration- and time-dependent manner [Fig 1A]. Increased expressed of *SPIB* and *GP2* were first evident at day 4 and peaked at day 7 following RANKL exposure [Fig 1B, data not shown]. Similar results were obtained when enteroids were grown in 3D configuration (data not shown).

Next we examined the timing of the RANKL effects on M cell formation. Two doses of RANKL on days 0 and 2 generated the highest expression of the M cell markers, *SPIB* and *GP2*, as assessed at varying times after RANKL exposure, whereas a single dose or three doses led to lower expression [Fig 1C]. In all dosing variations, a progressive decrease of both *SPIB* and *GP2* expression was observed at longer times after the final RANKL exposure [Fig 1C]. Together, these data show that RANKL stimulates expression of M cell associated transcripts in human enteroids in a concentration- and time-dependent manner that is maximally effective when added for a limited time early in culture.

**Fig 2:**
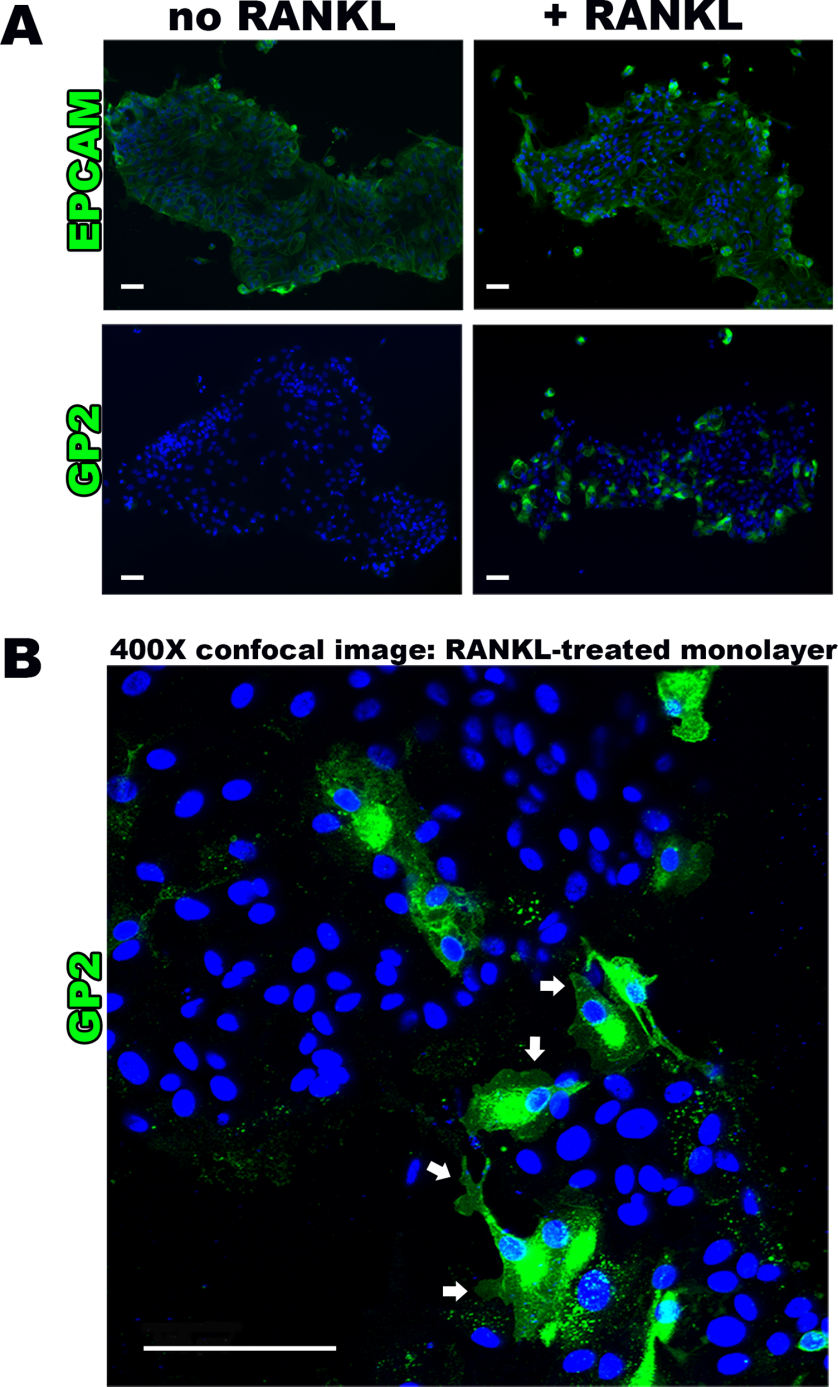
RANKL induces M cell surface marker presence and typical morphology. **(A)** Epifluorescense imaging (100X magnification) of human intestinal monolayers showing uniform expression of epithelial cell surface marker EPCAM in no RANKL and RANKL-treated monolayers, confirming epithelial origin. This is in comparison to M cell surface marker GP2 absence in no RANKL group versus positive GP2 staining in RANKL monolayer groups. **(B)** Confocal microscopy assessment (400X magnification) of immunostained RANKL treated monolayers showing membrane surface and cytoplasmic GP2 staining, demonstrating the characteristic M cell morphology of pseudopod-like projections (white arrows). Scale bar 100 μm.

The induction of M cell-specific transcripts was paralleled by increased protein expression, as shown by immunofluorescence staining for GP2 [Fig 2A]. Of the cells identified by DAPI nuclear staining, 36.0 ± 12.9% were positive for GP2 in the RANKL-treated group, compared to only 0.2 ± 0.4% in the untreated controls (*p*<0.005). The majority of the M cells were observed near the periphery of cell islands within the monolayers. Control staining for EPCAM, a ubiquitous epithelial cell surface marker, confirmed that the epithelial nature of majority of cells in the monolayer [Fig 2A]. Closer examination of the RANKL treated monolayer cultures by confocal microscopy revealed that the GP2-positive M cells when grown in 2D exhibited pseudopod-like cell surface structures extending from the membrane [Fig 2B]. Furthermore, the cells lacked the uniformity of epithelial cells seen by EPCAM staining of non-RANKL treated cultures [Fig 2B].

### Canonical WNT Primes Stem Cells for RANKL-Induced M Cell Formation

*In vitro* expansion of human enteroids is known to require canonical-WNT stimulation where R-spondin (RSPO) is indispensable, while WNT3a, and/or the GSK-3β inhibitor, CHIR99021 (GSKi) enhance robust growth [21,22,25]. RSPO, a potent inducer of the canonical-WNT pathway, significantly potentiated the time-dependent increase in *SPIB* and *GP2* expression when used alone even in the absence of RANKL [Fig 1B] [26]. To further assess the consequences of canonical-WNT on M cell differentiation, two experiments were performed. The first examined the effect of GSKi with and without RANKL, while the second determined the effect of various doses of WNT3a prior to passaging the cells in the presence or absence of RANKL.

**Fig 3:**
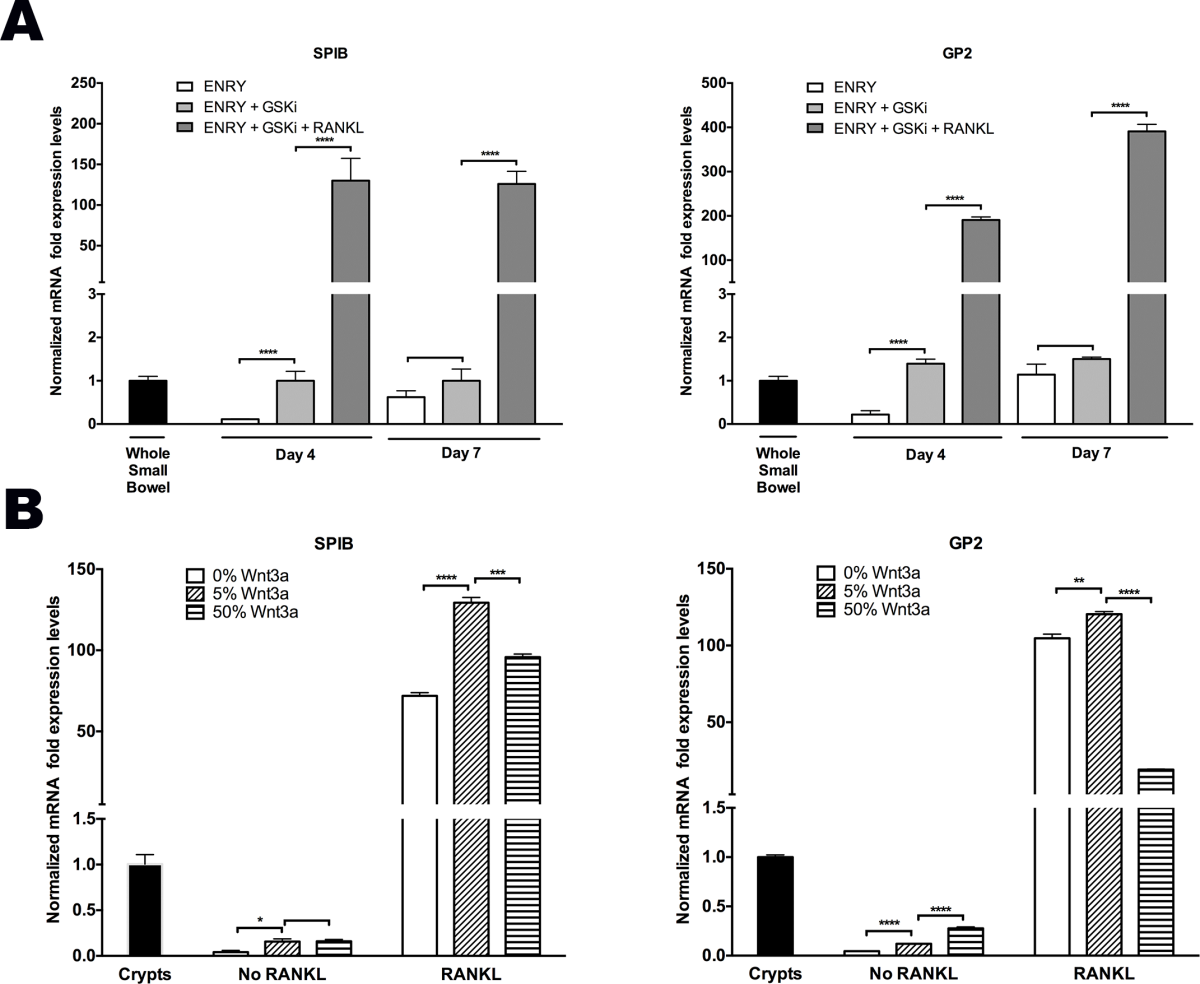
Canonical-WNT signal primes ISCs for RANKL induced M cell differentiation **(A)** Time course of *SPIB* and *GP2* peaked expression levels in monolayers treated with and without 200ng/mL RANKL. **(B)** Cells were treated with 0%, 5%, and 50% WNT3a-CM for 7 days, and on day 0 and 2 of the subsequent passage, cells were treated with ±200ng/mL RANKL and assessed 5 days later. All samples received ENRY complete medium and were assessed by quantitative PCR; all results normalized to *GAPDH* expression in human whole small bowel or crypts, as indicated. *p<0.*05*, **p<0.*01*, ***p<0.*001*, ****p<0.*0001*.

GSKi that was added on day 0 in the absence of RANKL increased *GP2* and *SPIB* transcripts by six-fold at an early time point (day 4) [Fig 3A]. By day 7, however, the ENRY only group (without GSKi) reached the same levels of *GP2* and *SPIB* expression, which suggests the possible role of RSPO synergism at later time points [Fig 3A]. As shown above, however, RANKL administration was required to achieve >100-fold enhanced ability for monolayers to form M cells [Fig 3A].

To further examine the consequences of WNT3a signaling, monolayers were grown with ENRY and treated for 7 days with various doses of WNT3a prior to passaging the cells in either the presence or absence of RANKL; cultures were assessed 5 days later. Monolayers treated with RANKL and 5% WNT3a-conditioned media (WNT3a-CM) showed the greatest M cell formation, as *SPIB* and *GP2* expression were 130- and 120-fold higher than controls, respectively [Fig 3B]. This is in comparison to the 0% WNT3a-CM group where *SPIB* and *GP2* mRNA levels increased 70 and 100-fold, and 50% WNT3a-CM group to 95- and 20-fold over controls, respectively [Fig 3B]. Without RANKL administration, all groups showed lower levels of expression compared to control samples. Taken together, these data suggest that the process of M cell differentiation is augmented through the canonical WNT signaling pathway, but that RANKL is required to induce a more robust differentiation process.

### Cultured M Cells Demonstrate Functionality with Increased Microparticle Uptake

**Fig 4:**
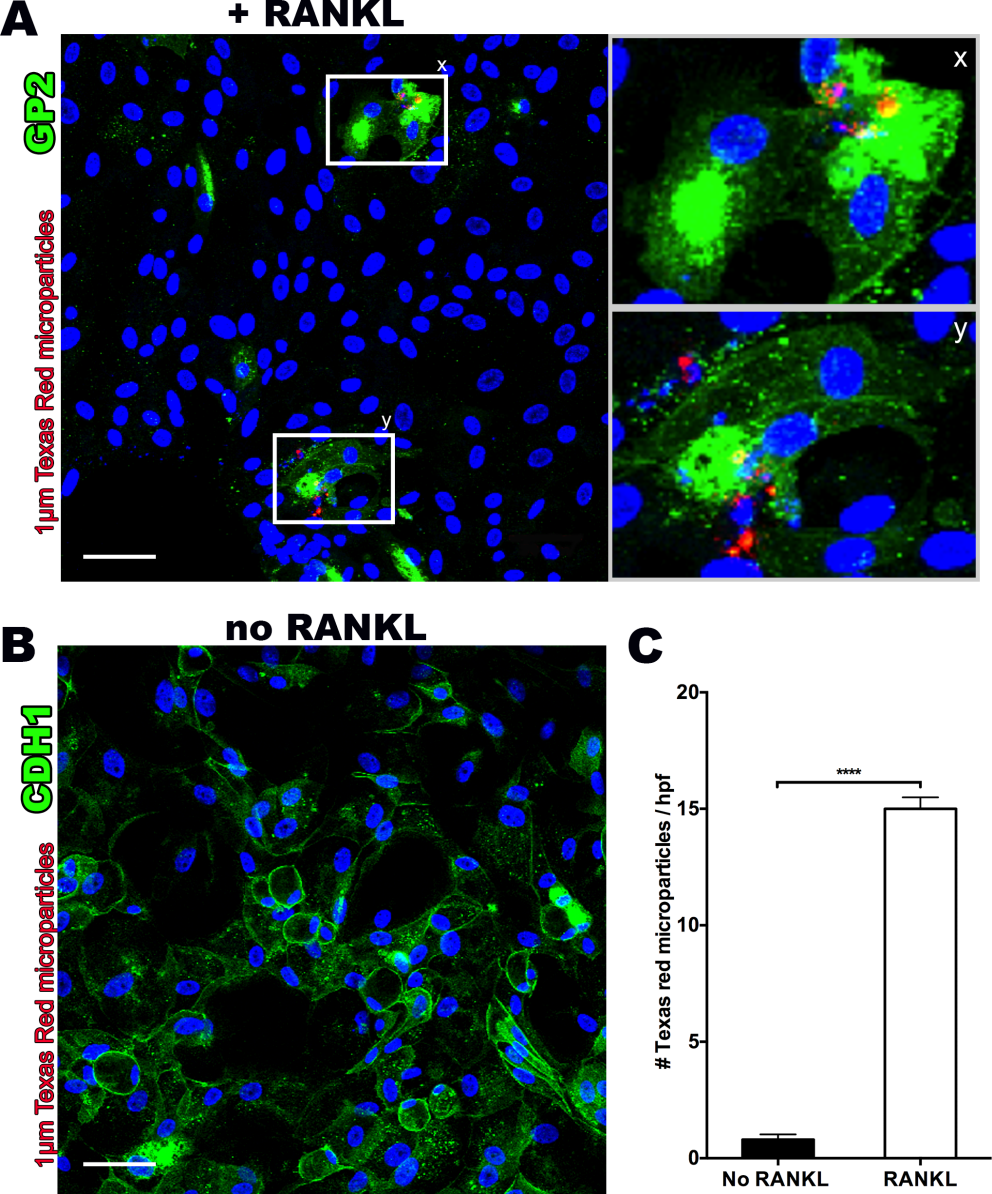
RANKL treated monolayers demonstrate functional M cells through uptake of fluorescent 1μm Texas Red latex microparticles. **(A)** Confocal microscopy z-stack assessment (400X magnification) showing colocalization of fluorescent 1μm Texas Red latex microparticles and GP2 positive stained M cells with enlarged sections [x,y] showing clumping of microparticles. **(B)** Confocal microscopy section (400X magnification) of non-RANKL monolayer stained for E-cadherin (CDH1), a cell surface marker that M cells lack, demonstrating uniform and ubiquitous staining, as well as lack of any Texas Red microparticle internalization. **(C)** RANKL treated monolayers show significantly increased numbers of retained fluorescent microparticles after washing, as assessed by three random high power field areas under confocal microscopy z-stack projections (400x). Scale bar 50 μm. ****p<0.*0001*.

M cells have a high capacity to endocytose and transcytose particulates [27]. Utilizing the same mechanisms that facilitate viral and bacterial transcytosis, M cells take up nanospheres as small as 20 nm, and microparticles as large as 10 μm [27]. The highest uptake rates are observed with particles of 1 μm diameter [28,29]. To explore the endocytic properties of *in vitro* derived human M cells, we incubated RANKL-treated monolayer cultures with fluorescent latex microparticles (1 μm) and performed confocal microscopy after 60–120 minutes. Microparticles were observed within GP2-positive cells using a Z-stack projection [Fig 4A]. By comparison, non-RANKL treated monolayers stained with the ubiquitous epithelial marker, E-cadherin, showed a lack of any microparticle uptake [Fig 4B]. Quantification by epifluorescence microscopy confirmed significantly greater (18-fold) microparticle uptake in RANKL-treated monolayer cultures compared to the non-RANKL treated group [Fig 4C].

### Preferential Uptake of *S*. Typhimurium into Cultured M Cells

**Fig 5:**
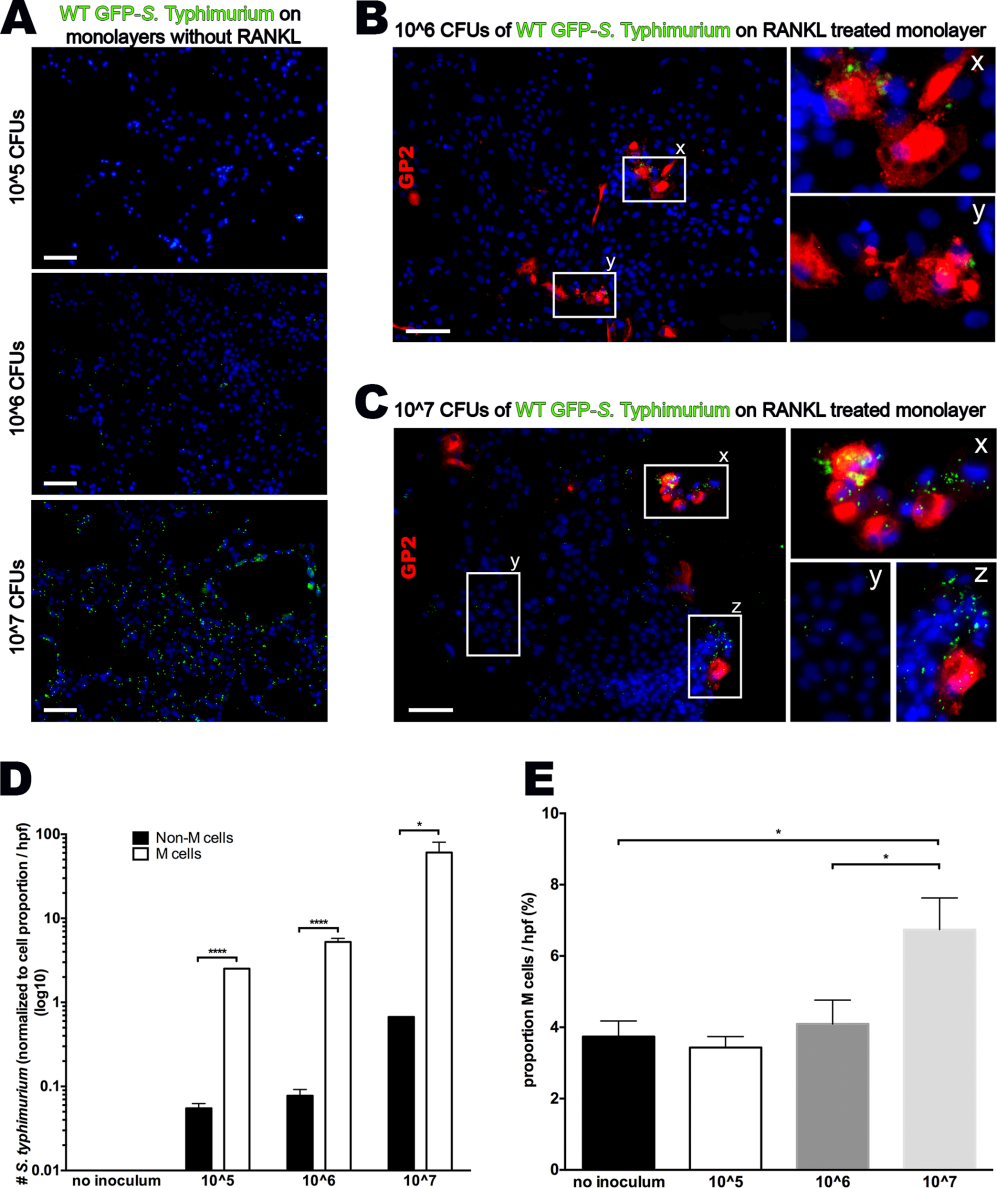
Cultured M cells preferentially uptake *S*. Typhimurium. **(A)** Epifluorescense imaging (100X) of monolayers without RANKL inoculated with 10^5, 10^6 and 10^7 CFUs of wild type GFP- *S*. Typhimurium. **(B)** Epifluorescense imaging (100X) of a RANKL treated monolayer inoculated with 10^6 CFUs of wild type GFP- *S*. Typhimurium showing uptake within GP2 stained cells, and respective magnification [x,y] showing clumping of many GFP-bacteria within the cell border. **(C)** Epifluorescense imaging (100X) of a RANKL treated monolayer inoculated with 10^7 CFUs of wild type GFP- *S*. Typhimurium showing increased overall GFP- *S*. Typhimurium uptake in clusters containing M cells [x,z] compared to non-M cell cluster [y]. **(D)** Normalized to cell density per high power field, *S*. Typhimurium bacteria were specifically associated with GP2-positive M cells in all inocula (log10 scale). **(E)** High inoculum *S*. Typhimurium associated with an increased number of M cells after only 60 minutes of bacterial presence; Scale bar 100μm;*p<0.*05*, **p<0.*01*, ***p<0.*001*, ****p<0.*0001*.

M cells function as a gateway for many bacterial and viral pathogens to initiate host infection. For example, *S*. Typhimurium exploits the phagocytic properties of M cells by preferentially adhering to and entering M cells in the intestinal mucosa [3,27]; however, this has never been shown with primary human cells To further explore the utility of our M cell model, we infected RANKL-treated and untreated monolayers with GFP-labeled *S*. Typhimurium for 1 h, washed the cultures, and incubated them for an additional hour with gentamicin to kill the remaining extracellular bacteria. Among RANKL untreated monolayers, there was very limited bacterial uptake in the 10^5 and 10^6 inocula, but a more generalized cellular uptake in the 10^7 inoculum [Fig 5A]. When compared to RANKL treated groups, bacteria were preferentially associated with GP2-positive M cells over a range of inocula [Fig 5B–5D]. Specifically, low (10^5 and 10^6) inocula non-M cells show a very low predilection for bacterial uptake, whereas, normalized to cell density, M cells were 50 and 65-fold more likely to contain *S*. Typhimurium [Fig 5B–5D]. In other words, virtually all *S*. Typhimurium bacteria were localized within M cells in these lower inocula. However, high inoculum (10^7) cultures show some bacterial co-localization both non-M cells and M cells. The key difference is that M cells in the high inoculum culture demonstrate a significantly higher density of bacterial sampling, containing many bacteria in each cell [Fig 5C]. Taken together, this data suggest that M cells preferentially endocystose *S*. Typhimurium bacteria, but at a critical concentration, invasion of non-M cells becomes possible.

### *S*. Typhimurium Induces M Cell Differentiation

Bacterial challenge, specifically with *S*. Typhimurium, in bovines was shown to induce rapid (<1 h) transdifferentiation of enterocytes to M cells in the presence of RANKL through the canonical-WNT pathway in a process mediated by the bacterial effector SopB [16]. While cultures infected with the lower inocula (10^5 and 10^6) had a similar proportion of M cells compared to uninfected control cultures, monolayers infected with the highest inoculum (10^7) showed a near doubling of the number of GP2+ cells within 1 h after infection [Fig 5E]. It shows for the first time that the transition is cell-autonomous without a requirement for other, non-epithelial cell types.

## Discussion

We have developed a robust human *in vitro* model that is enriched for M cells grown from crypts of the proximal small intestine. The cultured M cells demonstrate several of the characteristic mRNA transcripts and cell surface markers of M cells *in vivo*, as well as the capability to internalize microparticles and bacteria such as *S*. Typhimurium. To the best of our knowledge, this is the first report of a tractable human M cell model system.

We have previously shown that human intestinal enteroids can be grown from discarded surgical samples and propagated indefinitely by weekly passage [21,22,25]. Cells in this system retained the ability to differentiate into different epithelial lineages representative of crypt and villi, but they lacked M cell markers under typical culture conditions. This current study expands upon our previous work by demonstrating that functional M cells can be generated in culture in a robust manner that recapitulates human M cells formation and function *in vivo*.

Most M cell studies have focused on FAE, which overlies Peyer’s patches and is part of a continuous epithelial layer of the small intestine. In murine studies, M cell fate is dependent on RANKL stimulation, which induces the expression of *SpiB* through the WNT/**β**-catenin pathway [13,14,16]. Bacterial mediated mechanisms have also been shown to exploit the WNT/**β**-catenin pathway to induce rapid de novo M cell formation through a process of epithelial mesenchymal transition [16]. This study confirms prior knowledge that activation of the canonical-WNT pathway primes M cell differentiation; however, ultimate M cell differentiation is entirely dependent on RANKL through the NF-κB pathway [16]. Specifically, our data suggest significant induction of *SPIB* transcription activity even without RANKL stimulation in control groups receiving either RSPO alone or in combination with GSKi [Figs 1 and 3A]. However, exogenous RANKL is required to achieve optimal levels of *SPIB* activity to drive M cell generation. As expected, low dose WNT3a showed higher M cell marker expression compared to higher doses, likely because overstimulation with canonical WNT attenuates the more fully differentiated state [Fig 3B].

Consistent with work done in mice, our data shows that the *SPIB* expression peaks after 2 days of RANKL administration, with *GP2* levels peaking at day 4, and persisting through day 7 [Fig 3A, data not shown] [17]. We show that optimal RANKL delivery was at a concentration of 200 ng/mL administered at day 0 and day 2 only, while repeat dosing failed to further enhance M cell generation [Fig 1C].

In our monolayer cultures, we found evidence of deep pockets within M cells combined with pseudopod extensions [Fig 2B]. Additionally, we noticed a trail of GP2 staining adjacent to many of the M cells, and since GP2 selectively binds to the FimH component of the type I pili of *S*. Typhimurium and other pathogens, we expected to find bacteria adjacent to and within M cells [2]. This could possibly suggest GP2 shedding during the course of M cell movement toward the periphery, which may be related to typical movement along the FAE.

A crucial feature of M cells is their role as antigen sampling and transcytotic sites. Microparticles have been targeted to M cells in *in vivo* animal studies for vaccine, adjuvant therapy and drug delivery [27]. Multiple reports have characterized size parameters for uptake into M cells, showing they are capable of taking up particles from 50 nm to 10 μm, although particles in the 0.5–2 μm range are transcytosed most effectively [28,29]. We observed clumping of microparticles within M cells—suggesting that multiple particles may reside in the same phagosome. The ability to create M cells from human intestinal crypts that have functional uptake of microparticles may improve high-throughput methods for optimization of vaccine and drug delivery [27,30].

Many invasive bacteria cross the mucosal barrier often by exploiting the transcytotic ability of M cells and occasionally can be spread hematogenously [31–34]. *S*. Typhimurium not only invades M cells, but also through secretion of a type III effector protein named SopB, induces rapid differentiation of FAE enterocytes into M cells [16]. Interestingly, this occurs through inhibition of GSK3**β**, or the same **β**-catenin mediated mechanism as described above that we utilized to prime ISCs for M cell development [Fig 3A]. SopB was found to induce both the RANKL and the receptor RANK via WNT signaling, and was able to cause enterocyte transition within 60–180 minutes [16]. Specifically, SopB secreted by *S*. Typhimurium and other pathogens induce rapid transdifferentiation of enterocytes into M cells via EMT. However, in the bovine model, SopB-induced transition to M cells was limited to FAE crypts and was not observed in ordinary crypts [16]. In contrast, we found that increases in the density of *S*. Typhimurium delivery was associated with an increases in M cell number in non-FAE crypts isolated from the proximal bowel [Fig 5E]. The ability of proximal bowel crypts to be responsive to RANKL would suggest the presence of its receptor, RANK, or other important components of the signaling pathway in epithelium that might be capable of differentiating to M cells. Further experiments with increasing time of Salmonella presence would further characterize the time course of M cell transition that occurs with SopB presence.

The putative WNT source within the gut is more prominent within the niche located in the crypt base, and one might anticipate that since FAE are flat dome-like structures without the typical villus structure, that WNT signaling may be potent throughout [35]. However, the requirement of WNT/RANKL signaling to generate M cells would suggest that along the villus length in non-dome structures, M cells may differentiate at the crypt base and whether it retains M cell feature once these signals diminish would need to be addressed. Nevertheless, the lymphoid follicles in the gut are enriched with B cell expressing RANKL and their abundance and proximity likely accounts for the enrichment of M cell within the FAE. Additional assessments should include whether M cells generated from non-FAE crypts have a mesenchymal phenotype, and whether EMT or MET are common fates outside of the FAE during homeostatic and disease states such as during chronic inflammation or infection such as in environmental enteropathy will require further assessments using similar *in vitro* model systems.

## Material and Methods

### Ethics Statement

All human tissues used in this study were obtained from de-identified and discarded surgical specimens following clinical surgical pathology evaluation. This was approved by the University of California Los Angeles Institutional Review Board approved procurement and use of surgical samples, which waived the requirement for informed consent for tissues obtained from the UCLA Translational Pathology Core Laboratory (IRB #11–002504).

### Intestinal Crypt Isolation

Small intestinal crypts were isolated as previously described [21,23]. Briefly, crypts were resuspended in basic medium (Advanced Dulbecco's Modified Eagle Medium (ADMEM)/Ham's F12 (Invitrogen) with 2 mM GlutaMAX (Invitrogen), 10 mM HEPES (Invitrogen). Crypt yield was determined by microscopic counting.

### Cell Culture

A thin film of liquid type I collagen (Advanced BioMatrix, San Diego, CA) was plated onto the bottom surface of 48-well Nucleon Delta-treated cell culture plate (Thermo Scientific, Waltham, MA) at a concentration of 100 ng/mL, incubated for 30 minutes, and then removed by aspiration. Human crypts were plated at a density of 500 crypts per well. These were grown as monolayers in Basic Medium with antibiotic-antimycotic (Invitrogen), 1 mM N-Acetylcysteine (Sigma), 100 ng/ml recombinant murine Noggin (PeproTech), 50 ng/ml recombinant murine EGF (PeproTech), 1xN2 supplement (Invitrogen), 1xB27 supplement (Invitrogen), 1 μg/ml recombinant human R-spondin 1 (R&D Systems, Minneapolis, MN), 10 μM Y-27632 inhibitor (Stemgent), 1 mM recombinant human Jagged-1 (R&D Systems), 5 μM CHIR99021 (GSK-3β inhibitor) (Stemolecule). Alternatively, crypts were suspended within Matrigel at a concentration of 100 crypts per 25 μl of Matrigel and plated in 3D on the 48-well Nucleon Delta-treated cell culture plate.

### RANKL Stimulation

Cells were divided into groups and recombinant human RANKL (Peprotech, Rocky Hill, NJ) added at different concentrations (50–200 ng/mL). Controls were left without RANKL. Fresh culture medium, growth factors and RANKL were given at day 0 and replaced every 2 days until cultures were fixed for histologic or RNA analysis. Growth of two-dimensional monolayers and three-dimensional structures were assessed using inverted light microscopy.

### Subculturing of Human Monolayers

To examine the effect of WNT3a priming, a subset of monolayer cultures were treated with 0%, 5%, and 50% L-WNT3a conditioned medium (WNT3a CM), which was prepared as previously described, for 7 days [36]. After seven days of WNT3a-CM treatment, monolayers were digested using TrypLE (Life Technologies) at 37°C for three minutes. TrypLE was then quenched using 10% FBS in ADMEM/F12 and structures were mechanical split into small clusters of cells. In a 1:2 or 1:3 split, cell cluster were re-plated onto collagen-coated plates and split into 0%, 5% and 50% WNT3a-CM groups with and without 200 ng/mL RANKL.

### Microparticle Uptake

After 4 days of culture, 1x10^5^ 1 μm fluorescent-Texas Red polystyrene microparticles (Thermo Scientific) were added to monolayers and incubated for 60–120 minutes (Figs A-C in S1 File). Each well was then washed with PBS three times to remove non-adherent microparticles, and cultures were fixed with 3.4% formalin. Epifluorescence imaging was used to quantify microparticle uptake in three randomly chosen high power fields in RANKL treated and non-treated groups.

### Immunofluorescence and Confocal Microscopy

Monolayers cultured with and without latex microparticles were stained for Glycoprotein-2 (GP2) (MBL), E-cadherin (CDH1) (Dako), and Epithelial cell adhesion molecule (EPCAM) (Abcam). Epifluorescence microscopy was used to characterize differences between RANKL treated and non-treated groups. Confocal microscopy was used to characterize morphologic characteristics, as well as confirm z-stack co-localization of Texas Red latex microparticles within cells.

### *S*. Typhimurium Inoculation

After 4–9 days of culture, RANKL treated and untreated monolayers were inoculated with different inocula of GFP-labelled *S*. Typhimurium [37]. Antibiotics were removed by repeated washing before infections. Monolayers were then inoculated with 10^5, 10^6 and 10^7 CFU of GFP-labeled bacteria. After 1 h, plates were incubated with gentamicin for 15–60 mins, and washed three times with PBS. Monolayers were then fixed with 3.4% formalin for 5 mins.

Monolayers were stained for GP2 and DAPI, and epifluorescence imaging was used to quantify GFP-labeled Salmonella inside GP2-positive cells as well as in non-GP2 stained cells in three randomly chosen high power fields.

### RNA Analysis

At various time points, monolayer and three-dimensional cultures were harvested, and messenger RNA (mRNA) was isolated using Trizol Reagent (Life Technologies) method. Reverse transcriptase polymerase chain reaction (RT-PCR) was performed to characterize expression of *GP2*, *SPIB*, Chromogranin A (*CHGA*), and Glyceraldehyde-3-phosphate dehydrogenase (*GADPH*). RT-PCR reactions were performed on a Prism 7900 HT Sequence Detection System (Applied Biosystems). Cycle numbers were analyzed according to the comparative CT method using *GAPDH* as the internal calibrator and human intestinal crypts as the reference tissue [23].

### Statistical Analysis

Two-tailed unpaired student's t-test was used to compare results, and associated p-values are reported.

## Supporting Information

S1 File Texas red microparticles and Human ISC monolayers. **(Fig A)** Epifluorescense + light microscopy (25X) of a 2μL droplet of 1μm Texas red microparticles, and epifluorescense imaging (100X) of microparticles only pre- and post-washing demonstrating that microparticles can be washed away. **(Fig B)** Light microscopy (25X) of confluent monolayer of RANKL untreated and treated cells. **(Fig C)** Epifluorescense and light microscopy (100X) of RANKL-untreated and -treated monolayers incubated with Texas red microparticles and washed thrice showing greater microparticle presence in RANKL-treated group.
(TIF)

## Abbreviations

M Cells: Microfold cells
ISCs: Intestinal stem cells
*S*. Typhimurium: *Salmonella enterica* serovar Typhimurium
FAE: Follicle-associated epithelium
GP2: Glycoprotein 2
eGFP: enhanced green fluorescent protein
ENRY: **E**GF, **N**oggin, **R**-spondin, **Y**-27632 inhibitor
GSKi: GSK-3β inhibitor (CHIR99021)
RANKL: Receptor activator of NF-κB ligand

## References

[1] Amerongen HM, Weltzin R, Farnet CM, Michetti P, Haseltine WA, Neutra MR. Transepithelial transport of HIV-1 by intestinal M cells: a mechanism for transmission of AIDS. Journal of acquired immune deficiency syndromes (1999). 1991;4(8):760–5. 1856788

[2] Neutra M, Frey A, Kraehenbuhl J. Epithelial M cells: gateways for mucosal infection and immunization. Cell. 1996;86(L):345–8. 8756716 10.1016/s0092-8674(00)80106-3

[3] Gonzalez-Hernandez MB, Liu T, Payne HC, Stencel-Baerenwald JE, Ikizler M, Yagita H, et al Efficient norovirus and reovirus replication in the mouse intestine requires microfold (M) cells. Journal of virology. 2014 6;88(12):6934–43. 10.1128/JVI.00204-14 24696493 PMC4054386

[4] Amerongen HM, Weltzin R, Mack JA, Winner LS, Michetti P, Apter FM, et al M cell-mediated antigen transport and monoclonal IgA antibodies for mucosal immune protection. Annals of the New York Academy of Sciences. 1992;664:18–26. 1456648 10.1111/j.1749-6632.1992.tb39745.x

[5] Fotopoulos G, Harari A, Michetti P, Trono D, Pantaleo G, Kraehenbuhl J-P. Transepithelial transport of HIV-1 by M cells is receptor-mediated. Proceedings of the National Academy of Sciences of the United States of America. 2002;99(14):9410–4. 12093918 10.1073/pnas.142586899 PMC123154

[6] Fantini J, Yahi N, Chermann JC. Human immunodeficiency virus can infect the apical and basolateral surfaces of human colonic epithelial cells. Proceedings of the National Academy of Sciences of the United States of America. 1991;88(20):9297–301. 1718004 10.1073/pnas.88.20.9297 PMC52701

[7] Kerneis S. Conversion by Peyer’s Patch Lymphocytes of Human Enterocytes into M Cells that Transport Bacteria. Science. 1997 8 15;277(5328):949–52. 9252325 10.1126/science.277.5328.949

[8] Sato T, Vries RG, Snippert HJ, van de Wetering M, Barker N, Stange DE, et al Single Lgr5 stem cells build crypt-villus structures in vitro without a mesenchymal niche. Nature. Nature Publishing Group; 2009 5 14;459(7244):262–5. 10.1038/nature07935 19329995

[9] Buczacki SJ a, Zecchini HI, Nicholson AM, Russell R, Vermeulen L, Kemp R, et al Intestinal label-retaining cells are secretory precursors expressing Lgr5. Nature. 2013;495(7439):65–9. 10.1038/nature11965 23446353

[10] Jensen J, Pedersen EE, Galante P, Hald J, Heller RS, Ishibashi M, et al Control of endodermal endocrine development by Hes-1. Nature genetics. 2000;24(1):36–44. 10615124 10.1038/71657

[11] Yang Q, Bermingham NA, Finegold MJ, Zoghbi HY. Requirement of Math1 for secretory cell lineage commitment in the mouse intestine. Science (New York, NY). 2001;294(5549):2155–8. 10.1126/science.1065718 11739954

[12] Fre S, Huyghe M, Mourikis P, Robine S, Louvard D, Artavanis-Tsakonas S. Notch signals control the fate of immature progenitor cells in the intestine. Nature. 2005;435(7044):964–8. 15959516 10.1038/nature03589

[13] Knoop KA, Kumar N, Butler BR, Sakthivel SK, Taylor RT, Nochi T, et al RANKL is necessary and sufficient to initiate development of antigen-sampling M cells in the intestinal epithelium. Journal of immunology (Baltimore, Md: 1950). 2009;183(9):5738–47. 10.4049/jimmunol.0901563 PMC2922944 19828638

[14] Kanaya T, Hase K, Takahashi D, Fukuda S, Hoshino K, Sasaki I, et al The Ets transcription factor Spi-B is essential for the differentiation of intestinal microfold cells. Nature Immunology. 2012;13(8):729–36. 10.1038/ni.2352 22706340 PMC3704196

[15] Kobayashi a, Donaldson DS, Erridge C, Kanaya T, Williams IR, Ohno H, et al The functional maturation of M cells is dramatically reduced in the Peyer’s patches of aged mice. Mucosal immunology. Nature Publishing Group; 2013 9;6(5):1027–37. 10.1038/mi.2012.141 23360902 PMC3747980

[16] Tahoun A, Mahajan S, Paxton E, Malterer G, Donaldson DS, Wang D, et al Salmonella transforms follicle-associated epithelial cells into M cells to promote intestinal invasion. Cell host & microbe. 2012 11 15;12(5):645–56. 23159054 10.1016/j.chom.2012.10.009

[17] De Lau W, Kujala P, Schneeberger K, Middendorp S, Li VSW, Barker N, et al Peyer’s patch M cells derived from Lgr5(+) stem cells require SpiB and are induced by RankL in cultured “miniguts”. Molecular and cellular biology. 2012 9;32(18):3639–47. 10.1128/MCB.00434-12 22778137 PMC3430189

[18] Odero-Marah V a, Wang R, Chu G, Zayzafoon M, Xu J, Shi C, et al Receptor activator of NF-kappaB Ligand (RANKL) expression is associated with epithelial to mesenchymal transition in human prostate cancer cells. Cell research. 2008;18(8):858–70. 10.1038/cr.2008.84 18645583

[19] Min C, Eddy SF, Sherr DH, Sonenshein GE. NF-kB and epithelial to mesenchymal transition of cancer. Journal of Cellular Biochemistry. 2008;104(3):733–44. 10.1002/jcb.21695 18253935

[20] Zhou BP, Deng J, Xia W, Xu J, Li YM, Gunduz M, et al Dual regulation of Snail by GSK-3β-mediated phosphorylation in control of epithelial–mesenchymal transition. Nature Cell Biology. 2004;6(10):931–40. 15448698 10.1038/ncb1173

[21] Lahar N, Lei NY, Wang J, Jabaji Z, Tung SC, Joshi V, et al Intestinal subepithelial myofibroblasts support in vitro and in vivo growth of human small intestinal epithelium. PloS one. 2011;6(11):e26898 10.1371/journal.pone.0026898 22125602 PMC3219641

[22] Wang F, Scoville D, He XC, Mahe MM, Box A, Perry JM, et al Isolation and characterization of intestinal stem cells based on surface marker combinations and colony-formation assay. Gastroenterology. 2013;145(2):383–95. 10.1053/j.gastro.2013.04.050 23644405 PMC3781924

[23] Jabaji Z, Brinkley GJ, Khalil H a, Sears CM, Lei NY, Lewis M, et al Type I collagen as an extracellular matrix for the in vitro growth of human small intestinal epithelium. PloS one. 2014;9(9):e107814 10.1371/journal.pone.0107814 25222024 PMC4164635

[24] Terahara K, Yoshida M, Igarashi O, Nochi T, Pontes GS, Hase K, et al Comprehensive gene expression profiling of Peyer’s patch M cells, villous M-like cells, and intestinal epithelial cells. Journal of immunology (Baltimore, Md: 1950). 2008;180(12):7840–6. 10.4049/jimmunol.180.12.7840 18523247

[25] Jabaji Z, Sears CM, Brinkley GJ, Lei NY, Joshi VS, Wang J, et al Use of Collagen Gel as an Alternative Extracellular Matrix for the In Vitro and In Vivo Growth of Murine Small Intestinal Epithelium. Tissue Engineering: Part C. 2013;19(12):961–9. 10.1089/ten.tec.2012.0710 PMC3833386 23566043

[26] De Lau WB, Snel B, Clevers HC. The R-spondin protein family. Genome Biology. 2012;13(3):242 10.1186/gb-2012-13-3-242 22439850 PMC3439965

[27] Ermak T, Giannasca P. Microparticle targeting to M cells. Advanced drug delivery reviews. 1998 12 1;34(2–3):261–83. 10837681 10.1016/s0169-409x(98)00043-x

[28] Desai MP, Labhasetwar V, Amidon GL, Levy RJ. Gastrointestinal uptake of biodegradable microparticles: Effect of particle size. Pharmaceutical Research. 1996;13(12):1838–45. 8987081 10.1023/a:1016085108889

[29] Walter E, Dreher D, Kok M, Thiele L, Kiama SG, Gehr P, et al Hydrophilic poly(dl-lactide-co-glycolide) microspheres for the delivery of DNA to human-derived macrophages and dendritic cells. Journal of Controlled Release. 2001 9 11;76(1–2):149–68. 11532321 10.1016/s0168-3659(01)00413-8

[30] Pappo J, Ermak TH. Uptake and translocation of fluorescent latex particles by rabbit Peyer’s patch follicle epithelium: a quantitative model for M cell uptake. Clinical and experimental immunology. 1989;76(1):144–8. 2661061 PMC1541725

[31] Kraehenbuhl J-P, Neutra MR. Epithelial M cells: differentiation and function. Annual Review of Cell and Developmental Biology. 2000;16(1):301–32. 10.1146/annurev.cellbio.16.1.301 11031239

[32] Apter FM, Michetti P, Winner LS, Mack JA, Mekalanos JJ, Neutra MR. Analysis of the roles of antilipopolysaccharide and anti-cholera toxin immunologlubulin A (IgA) antibodies in protection against Vibrio cholerae and cholera toxin by use of monoclonal IgA antibodies in vivo. Infection and Immunity. 1993;61(12):5279–85. 8225601 10.1128/iai.61.12.5279-5285.1993 PMC281312

[33] Walker RI, Schmauder-Chock EA, Parker JL, Burr D. Selective association and transport of Campylobacter jejuni through M cells of rabbit Peyer’s patches. Canadian journal of microbiology. 1988;34(10):1142–7. 3196964 10.1139/m88-201

[34] Grützkau a, Hanski C, Hahn H, Riecken EO. Involvement of M cells in the bacterial invasion of Peyer’s patches: a common mechanism shared by Yersinia enterocolitica and other enteroinvasive bacteria. Gut. 1990;31(9):1011–5. 2210445 10.1136/gut.31.9.1011 PMC1378659

[35] Vallin J, Thuret R, Giacomello E, Faraldo MM, Thiery JP, Broders F. Cloning and Characterization of Three Xenopus Slug Promoters Reveal Direct Regulation by Lef/β-Catenin Signaling. Journal of Biological Chemistry. 2001;276(32):30350–8. 11402039 10.1074/jbc.M103167200

[36] Willert K, Brown JD, Danenberg E, Duncan AW, Weissman IL, Reya T, et al Wnt proteins are lipid-modified and can act as stem cell growth factors. Nature. 2003;423(6938):448–52. 12717451 10.1038/nature01611

[37] Ma L, Zhang G, Doyle MP. Green Fluorescent Protein Labeling of Listeria, Salmonella, and Escherichia coli O157:H7 for Safety-Related Studies. PLoS ONE. 2011;6(4):e18083 10.1371/journal.pone.0018083 21483738 PMC3070700

