# Supplementary figures and images for: Development of Functional Microfold (M) Cells from Intestinal Stem Cells in Primary Human Enteroids
Source: PLoS One. 2016 Jan 28;11(1):e0148216. doi: 10.1371/journal.pone.0148216 (PMC4731053; doi:10.1371/journal.pone.0148216)

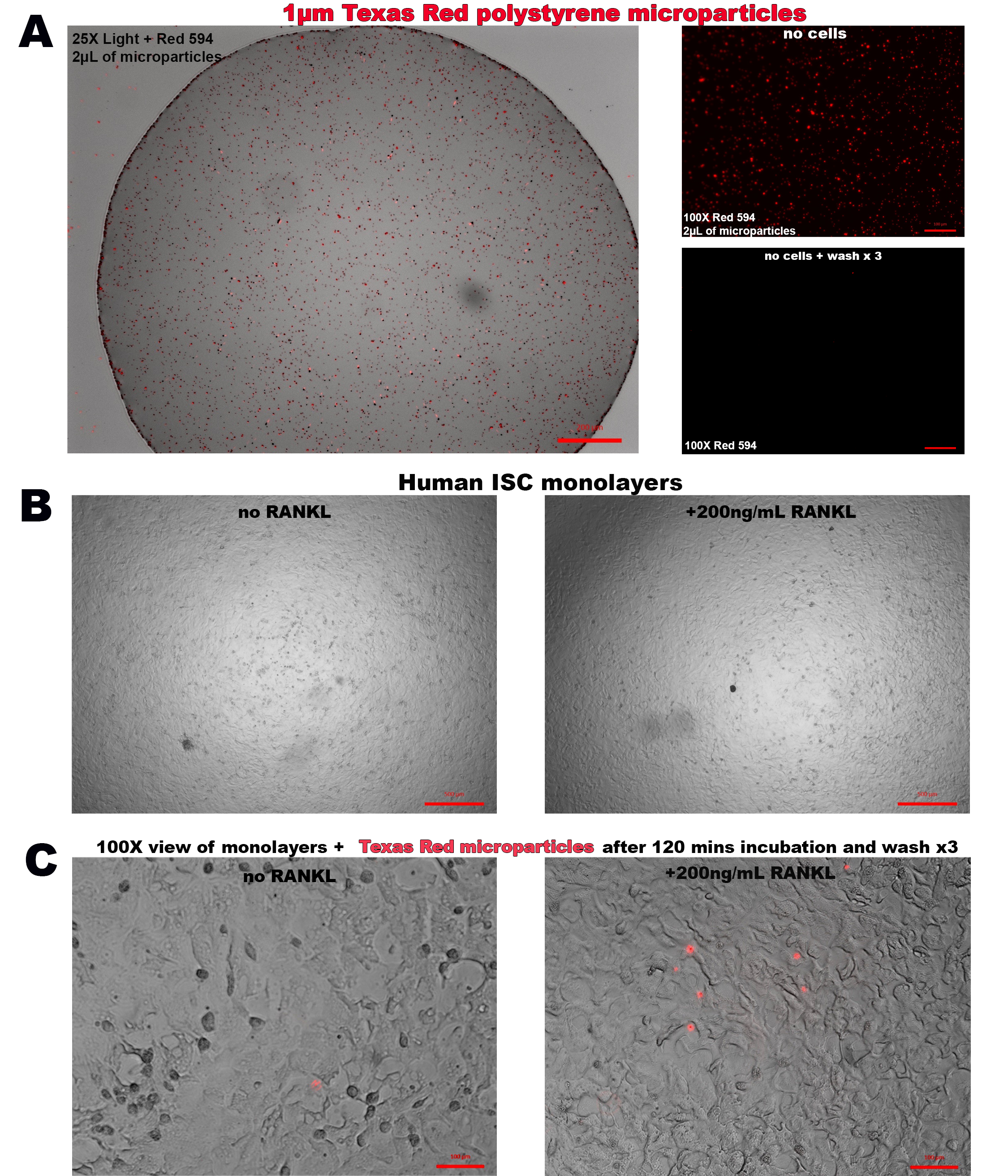

Supplement: S1 File — (Fig A) Epifluorescense + light microscopy (25X) of a 2μL droplet of 1μm Texas red microparticles, and epifluorescense imaging (100X) of microparticles only pre- and post-washing demonstrating that microparticles can be washed away. (Fig B) Light microscopy (25X) of confluent monolayer of RANKL untreated and treated cells. (Fig C) Epifluorescense and light microscopy (100X) of RANKL-untreated and -treated monolayers incubated with Texas red microparticles and washed thrice showing greater microparticle presence in RANKL-treated group. (TIF) [file pone.0148216.s001.tif]
